# Association of Pre‐ and Postdiagnosis Physical Activity, Promotion and Maintenance With Lung Cancer Survival: A Nationwide Cohort Study

**DOI:** 10.1002/jcsm.70092

**Published:** 2025-10-17

**Authors:** Yeon Wook Kim, Kyeong Im Kwak, A‐Reum Choi, Eung Joo Park, Brian J. Lee, Yeon Joo Lee, Choon‐Taek Lee

**Affiliations:** ^1^ Division of Pulmonary and Critical Care Medicine, Department of Internal Medicine Seoul National University College of Medicine Seoul Republic of Korea; ^2^ Division of Pulmonary and Critical Care Medicine, Department of Internal Medicine Seoul National University Bundang Hospital Seongnam Republic of Korea; ^3^ Big Data Center Seoul National University Bundang Hospital Seongnam Republic of Korea; ^4^ Emeritus Professor, Seoul National University College of Medicine Seoul Republic of Korea

**Keywords:** all‐cause mortality, lung cancer, physical activity, survivorship

## Abstract

**Background:**

Physical activity (PA) is important for improving life expectancy and is suggested as a prognostic factor for various diseases. However, the association between PA and mortality outcomes in survivors of lung cancer remains unclear. Therefore, this study aimed to determine the association of PA levels, including changes before and after diagnosis, with mortality outcomes among survivors of lung cancer.

**Methods:**

We conducted a nationwide cohort study involving 23 257 individuals diagnosed with lung cancer between 1 January 2010 and 31 December 2016, who attended the National Health Screening Program within 2 years before and after diagnosis. Pre‐ and postdiagnosis leisure‐time PA levels and changes in PA were assessed using self‐administered questionnaires. Individuals who reported engaging in moderate‐intensity exercise ≥ 5 days/week or vigorous‐intensity exercise ≥ 3 days/week were classified as physically active. The amount of PA was calculated as the metabolic equivalent of task (MET)‐min/week for each individual. All participants were followed from the date of diagnosis to 31 December 2022, for the outcome of mortality.

**Results:**

During the follow‐up period of 165 344.0 person‐years, 9094 deaths occurred (6633 lung cancer‐specific and 2461 non–lung cancer deaths). Multivariable analyses revealed that both pre‐ and postdiagnosis PA were associated with significantly reduced risk of all‐cause (aHR = 0.92, 95% CI = 0.88–0.97 for prediagnosis and aHR = 0.85, 95% CI = 0.81–0.89 for postdiagnosis) and lung cancer‐specific (aHR = 0.93, 95% CI = 0.88–0.99 for prediagnosis and aHR = 0.89, 95% CI = 0.84–0.94 for postdiagnosis) mortality compared with inactivity. Significant dose–response relationships were observed between PA levels and mortality risk reduction. Compared with individuals who were consistently inactive before and after diagnosis, significant mortality risk reduction was seen in those who maintained PA (aHR = 0.77, 95% CI = 0.71–0.83 for all‐cause and aHR = 0.81, 95% CI = 0.75–0.89 for lung cancer‐specific mortality) and those who promoted PA after lung cancer diagnosis (aHR = 0.91, 95% CI = 0.85–0.96 for all‐cause and aHR = 0.94, 95% CI = 0.88–1.00 for lung cancer‐specific mortality). However, individuals who were active before diagnosis but became inactive after diagnosis showed no significant difference in survival outcomes compared with those who were consistently inactive.

**Conclusions:**

Both pre‐ and postdiagnosis PA are associated with reduced mortality in survivors of lung cancer in a dose–response manner. Maintenance and promotion of PA after diagnosis are key to achieving optimal benefits in overall survival.

**Abbreviations**
BMI: body mass index
CCI: Charlson Comorbidity Index
CI: confidence interval
HR: hazard ratio
ICD‐10: International Statistical Classification of Diseases and Related Health Problems 10th edition
MET: metabolic equivalent of task
NHIS: National Health Insurance Service
PA: physical activity
PLCO: Prostate, Lung, Colorectal, and Ovarian Cancer Screening Trial
SD: standard deviation
WHO: World Health Organization

## 1 Background

Lung cancer is the second most commonly diagnosed cancer type, and the leading cause of cancer‐related death globally [1]. Owing to the latency of symptom presentation, patients are commonly diagnosed at an advanced stage, leading to poor survival outcomes [1, 2]. Recently, with the widespread implementation of lung cancer screening using low‐dose computed tomography, improvements in early detection, and advances in treatment modalities, the global population of lung cancer survivors is increasing. In Korea, 5‐year survival rates have reached 78.5% for localized and 48.4% for regional lung cancers [3], leading to more than 120 000 individuals living with or beyond lung cancer in 2021 [4]. With improving prognosis, a growing number of patients with lung cancer now have sufficient lifespans to warrant consideration of non‐cancer causes of mortality [5, 6]. Therefore, in addition to cancer treatment modalities, the importance of identifying modifiable lifestyle factors associated with overall survival is expected to increase. Promoting a healthy lifestyle that can complement therapeutic approaches to further reduce cancer‐related mortality while concurrently lowering the risk of death from other causes is crucial for improving all‐cause mortality among survivors of lung cancer [7].

Physical activity (PA) is a key determinant of health and longevity strongly recommended by the World Health Organization (WHO) and international guidelines [8, 9]. It has been widely investigated as a prognostic factor for all‐cause mortality in various cancer types, given its known beneficial effects in reducing comorbidities and improving physiological function. Epidemiological studies have demonstrated that higher levels of PA are associated with improved overall survival in several malignancies, including breast, colon and prostate cancers [10, 11, 12, 13]. In addition, PA has been linked to reduced cancer‐specific mortality through biological mechanisms such as modulation of inflammation and immune function, which enhance treatment efficacy and reduce recurrence risk. PA also contributes to improved quality of life and alleviates treatment‐related symptoms such as fatigue and depression, further supporting favourable cancer‐specific outcomes [14, 15].

In lung cancer, although several studies, including a recent pooled analysis, have reported improved survivorship associated with PA levels [16, 17, 18, 19], other cohort studies have demonstrated nonsignificant associations [10]. Importantly, most of the existing evidence is based on small sample sizes and single time‐point assessments of pre‐ or postdiagnosis PA and shows inconsistent findings regarding its association with both all‐cause and cancer‐specific mortality [19]. The most recent guidelines from the American College of Sports Medicine (ACSM) and the American Cancer Society (ACS) concluded that a definitive association between PA and cancer survival can be established for cancer types with sufficient data covering all four associations: pre‐ and postdiagnosis PA, and all‐cause and cancer‐specific mortality [13, 20]. As such, the current body of evidence remains limited and inconclusive for lung cancer. Furthermore, the lack of data incorporating both pre‐ and postdiagnosis PA hampers the evaluation of the impact of promoting and maintaining PA after diagnosis on survival outcomes—an important consideration for developing practical recommendations across the continuum of lung cancer care.

Large‐scale investigations that address previous limitations are needed to fill the research gap and provide convincing evidence on whether and how pre‐ and postdiagnosis PA, as well as changes between these phases, influence survival. To this end, we conducted a nationwide, population‐based cohort study in Korea to evaluate the association between PA and long‐term survival among patients with lung cancer. To provide comprehensive evidence across the lung cancer care continuum, we aimed to examine the distinct associations of pre‐ and postdiagnosis PA, including changes in PA levels, using the time of diagnosis as an anchor point. Separate analyses were conducted for all‐cause and cancer‐specific mortality.

## 2 Methods

### 2.1 Data Source

We constructed a population‐based cohort using data from the South Korean National Health Insurance Service (NHIS) database. South Korea has a single‐payer universal health system, which provides mandatory health insurance and comprehensive medical coverage for more than 97% of the population. The NHIS database maintains the national records of all demographic characteristics, diagnostic codes per the International Statistical Classification of Diseases and Related Health Problems, 10th edition (ICD‐10), inpatient and outpatient visits, medical procedures, and pharmaceutical prescriptions [21, 22]. In addition, the NHIS provides biennial health screening for the following: (1) all employees, including self‐employed, insured individuals aged ≥ 20 years and (2) all individuals aged ≥ 40 years regardless of their employment status. The screening programme includes a self‐administered questionnaire on lifestyle behaviours such as leisure‐time PA, smoking, alcohol intake, anthropometric measurements and laboratory tests using blood and urine samples [23]. The NHIS database provides anonymized data for research purposes along with death records, including the cause of death, integrated from the Statistics Korea database, in adherence with strict confidentiality guidelines. This database has been used in large epidemiological studies and is widely validated [22, 24, 25].

### 2.2 Study Population

This study initially included 173 508 individuals newly diagnosed with lung cancer between 1 January 2010 and 31 December 2016. Lung cancer diagnosis was identified using registered diagnosis codes (ICD‐10, C33 and C34) with a specific insurance registration code for lung cancer (V193 or V194). In Korea, once a patient receives the V193 or V194 code, they are registered in the National Cancer Registry with a specific code indicating that they are eligible for special insurance benefits owing to the cancer diagnosis. Once a patient receives a V‐code, it is documented in their medical records and used in subsequent claims. Therefore, lung cancer diagnosis based on this registration code is considered highly reliable [25]. To evaluate the association of PA levels, including changes before and after diagnosis, with mortality outcomes among survivors of lung cancer, we included individuals who attended NHIS‐provided health screening within 2 years before (prediagnosis) and after (postdiagnosis) the date of diagnosis. After excluding individuals with missing demographic data, 23 257 individuals were included in the analysis (Figure S1; a comparison between included and excluded individuals is provided in Table S1). All participants were followed up from baseline (date of lung cancer diagnosis) to the date of death or the end of the study period (31 December 2022), whichever occurred first. The study design was approved by the Institutional Review Boards of the Seoul National University Bundang Hospital (No. X‐2203‐747‐901) and the NHIS (No. NHIS‐2023‐1‐054). The requirement for written informed consent was waived because all data were anonymised.

### 2.3 Definition and Measure of Variables

Leisure‐time PA was assessed as the primary exposure, as it is most closely related to individual lifestyle behaviour and is known to significantly decrease in patients with cancer, thereby representing a key target for behavioural interventions [12]. PA levels were collected using self‐administered questionnaires adopted by the NHIS, with modifications from the short‐form International Physical Activity Questionnaire developed by the WHO. The validity of this instrument has been established in the Korean population [26]. It has also been widely utilized to assess PA levels in epidemiologic studies involving both the general population and individuals with various diseases, including cancer [27, 28, 29, 30].

The survey used a 7‐day recall method and included three questions that addressed the usual frequency (days per week) of (1) light‐intensity exercise for at least 30 min (e.g., walking at a slow or leisurely pace); (2) moderate‐intensity exercise for at least 30 min (e.g., brisk walking, slow cycling or tennis doubles); and (3) vigorous‐intensity exercise for at least 20 min (e.g., jogging or running, cycling > 15 km/h, climbing briskly up a hill or participating in an aerobics class). According to the contemporaneous ACSM guidelines in effect at the time that this survey was adopted, which determined PA recommendations based on frequency per week [31], individuals who reported performing moderate‐intensity exercise ≥ 5 days or vigorous‐intensity exercise ≥ 3 days were deemed physically active. In addition, we calculated each individual's minimum estimated energy expenditure in metabolic equivalent task per week (MET‐min/week), based on the frequency of PA at each intensity level and the predefined minimum duration per session. MET‐min ratings of 3.0, 5.0 and 8.0 were assigned for light‐intensity, moderate‐intensity and vigorous‐intensity activities based on the Compendium of Physical Activity [32]. Total pre‐ and postdiagnosis MET‐min/week were calculated by summing the products of frequency, intensity and minimum duration. For each individual, pre‐ and postdiagnosis PA statuses (active or inactive) and amounts (in MET‐min/week), as well as changes in PA status before and after diagnosis, were determined.

Other baseline demographic data were collected from the most recent health screening results within 2 years of lung cancer diagnosis. Income levels were dichotomized into quintiles of annual income derived from insurance premiums. The average daily amount of alcohol consumed was calculated based on the weekly frequency of alcohol consumption and the amount of alcohol consumed per occasion. Participants were classified as nondrinkers, light‐to‐moderate drinkers (< 30 g/day) and heavy drinkers (≥ 30 g/day). Comorbidities were defined based on ICD‐10 codes registered within 1 year of the screening date and summarized according to the Charlson Comorbidity Index (CCI) [33].

### 2.4 Evaluation of Outcomes

The primary outcome of this study was all‐cause mortality, defined as death from any cause after lung cancer diagnosis. The secondary outcomes included lung cancer‐specific mortality and deaths from other causes. Vital status, date of death, and cause of mortality were determined through linkage with the Statistics Korea database using anonymized identification codes, with follow‐up completed on 31 December 2022. Causes of death were classified based on ICD‐10 codes, determined through medical record review. This database, supplemented by the Korea Statistics Promotion Institute, serves as the basis for national mortality statistics. Deaths due to lung cancer were identified using ICD‐10 codes C33 and C34, while all other causes were categorized as non–lung cancer mortality. Associations of pre‐ and postdiagnosis PA (status, amount, and change in status before and after diagnosis) with mortality outcomes were evaluated.

### 2.5 Statistical Analysis

The characteristics of the study population are presented as mean (SD) or median (IQR) values for continuous variables and proportions (%) for categorical variables. Baseline characteristics were compared between groups classified by pre‐ and postdiagnosis PA status, using the analysis of variance for continuous variables and *χ* ^2^ tests for categorical variables. Multivariable Cox proportional hazards regression models, with setting the date of lung cancer diagnosis as the baseline, were used to estimate hazard ratios (HRs) and 95% confidence intervals (CIs) for the associations of pre‐ and postdiagnosis PA (including changes between pre‐ and postdiagnosis), amount of PA (MET‐min/week levels) stratified by quartiles, with overall, lung cancer‐specific and non–lung cancer mortality. The minimally clinically important difference (MCID) for change in PA (MET/−min/week) was estimated using an outcome‐based approach anchored to all‐cause mortality.

With the inclusion of covariates with *p* values < 0.2 in univariable analyses, the final multivariable models were adjusted for age, sex, body mass index (BMI), smoking status, smoking pack‐year, income (categorical; in quintiles), alcohol consumption (categorical; non‐, light‐to‐moderate, heavy drinkers), CCI and received treatment (surgery, radiotherapy and chemotherapy) of diagnosed lung cancer. Kaplan–Meier curves for all‐cause and cause‐specific mortality were constructed and compared between groups determined by pre‐ and postdiagnosis PA using log‐rank tests, with the evaluation of the proportional hazards assumption for all analyses. Sensitivity analyses were conducted by excluding deaths that occurred during the first 2‐year follow‐up to reduce the probability of reverse causation [10, 12]. Finally, subgroup analyses were performed to evaluate the associations in long‐term (≥ 5 years) survivors of lung cancer. For all analyses, two‐sided *p* values < 0.05 were considered statistically significant. All analyses were performed using SAS version 9.4 (SAS Institute Inc.) and R Version 3.5.3 (http://www.R‐project.org).

## 3 Results

### 3.1 Characteristics of the Study Population

Table 1 describes the demographic characteristics of all the participants stratified by pre‐ and postdiagnosis PA status. Compared to individuals who remained consistently inactive (inactive to inactive) or did not maintain PA (active to inactive) after lung cancer diagnosis, individuals who promoted to becoming active after being inactive (inactive to active) or remained consistently active (active to active) tended to be male, younger and were more likely to undergo surgical treatment for lung cancer.

**TABLE 1:** Demographic characteristics of the study population.

Characteristics	Pre‐ and postdiagnosis physical activity status				*p*
	Inactive → inactive (*n* = 13 528)	Active → inactive (*n* = 2966)	Inactive → active (*n* = 4146)	Active → active (*n* = 2617)	
Age, years, mean ± SD	64.6 ± 10.2	64.5 ± 9.5	62.0 ± 9.3	63.0 ± 9.0	< 0.001
Sex, *n* (%)					< 0.001
Male	7765 (57.4)	1828 (61.6)	2570 (62.0)	1773 (67.7)	
Female	5763 (42.6)	1138 (38.4)	1576 (38.0)	844 (32.3)	
BMI, kg/m^2^, mean ± SD	23.6 ± 3.2	23.6 ± 3.0	23.6 ± 3.0	23.8 ± 2.8	0.02
Smoking status					< 0.001
Never‐smoker	7013 (51.8)	1467 (49.5)	1998 (48.2)	1173 (44.8)	
Ex‐smoker	5663 (41.9)	1352 (45.6)	1997 (48.2)	1343 (51.3)	
Current smoker	852 (6.3)	147 (5.0)	151 (3.6)	101 (3.9)	
Pack‐years smoked, mean ± SD	15.5 ± 23.2	15.0 ± 21.2	15.2 ± 21.8	14.8 ± 20.1	0.40
Income in quartiles, *n* (%)					< 0.001
1st quintile (highest)	4383 (32.4)	1013 (34.2)	1334 (32.2)	996 (38.1)	
2nd quintile	2900 (21.4)	623 (21.0)	893 (21.5)	534 (20.4)	
3rd quintile	2074 (15.3)	417 (14.1)	671 (16.2)	349 (13.3)	
4th quintile	1683 (12.4)	379 (12.8)	534 (12.9)	293 (11.2)	
5th quintile (lowest)	2488 (18.4)	534 (18.0)	714 (17.2)	445 (17.0)	
Alcohol consumption, *n* (%)					< 0.001
Non‐drinker	11 595 (85.7)	2508 (84.6)	3535 (85.3)	2088 (79.8)	
Light‐to moderate drinker	1509 (11.2)	347 (11.7)	483 (11.6)	438 (16.7)	
Heavy drinker	424 (3.1)	111 (3.7)	128 (3.1)	91 (3.5)	
Physical activity amount, mean ± SD					
Prediagnosis MET‐min/week	316.1 ± 311.6	1516.6 ± 383.9	406.2 ± 328.1	1585.9 ± 578.8	< 0.001
Postdiagnosis MET‐min/week	364.3 ± 316.0	448.2 ± 329.2	1559.0 ± 582.1	1631.8 ± 585.0	< 0.001
Charlson comorbidity index					< 0.001
1–2	8154 (60.3)	1799 (60.7)	2656 (64.1)	1662 (63.5)	
3–4	4057 (30.0)	886 (29.9)	1150 (27.7)	742 (28.4)	
≥ 5	1317 (9.7)	281 (9.5)	340 (8.2)	213 (8.1)	
Received treatment, *n* (%)					
Surgery	8760 (64.8)	2021 (68.1)	2995 (72.2)	1936 (74.0)	< 0.001
Radiotherapy	2671 (19.7)	594 (20.0)	802 (19.3)	422 (16.1)	< 0.001
Systematic therapy	1229 (9.1)	308 (10.4)	409 (9.9)	226 (8.6)	0.052
Follow‐up duration since diagnosis, years, mean ± SD	7.0 ± 3.2	6.9 ± 3.2	7.4 ± 3.0	7.6 ± 2.9	< 0.001

Abbreviations: BMI, body mass index; MET, metabolic equivalent of task; SD, standard deviation.

### 3.2 Association With Pre‐ and Postdiagnosis PA and Mortality Risk

**FIGURE 1:**
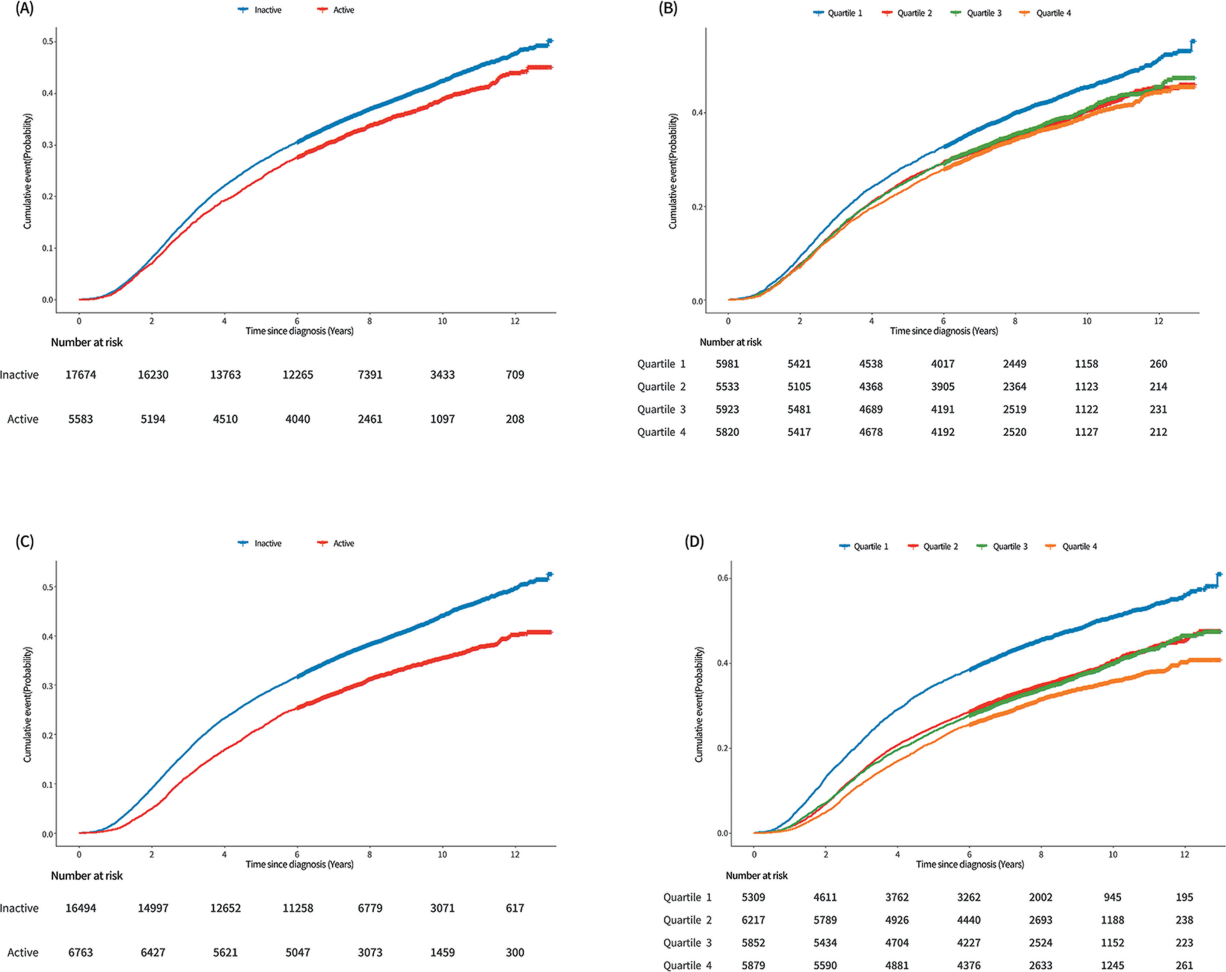
Cumulative incidence of all‐cause mortality in survivors of lung cancer by prediagnosis (A: status, B: amount [MET‐min/week] in quartiles) and postdiagnosis physical activity (C: status, D: amount [MET‐min/week] in quartiles).

During a follow‐up period of 165 344.0 person‐years, 9094 all‐cause mortalities (6633 lung cancer‐specific deaths and 2461 non–lung cancer deaths) occurred. Table 2 depicts the risk of overall, lung cancer‐specific and non–lung cancer mortality according to pre‐diagnosis PA measures. Compared with individuals who were physically inactive, physically active individuals had a significantly reduced mortality risk after adjustments for possible confounding variables (adjusted HR [aHR] = 0.92, 95% CI = 0.88–0.97 for all‐cause; aHR = 0.93, 95% CI = 0.88–0.99 for lung cancer‐specific; and aHR = 0.89, 95% CI = 0.81–0.98 for non–lung cancer mortality). When stratified by quartiles according to the total amount of pre‐diagnosis PA (in MET‐min/week), there was a significant trend towards decreasing risk of mortality with increasing amounts of pre‐diagnosis PA (Table 2 and Figures 1A,B, S2 and S3).

**TABLE 2:** Association between prediagnosis physical activity and mortality in survivors of lung cancer.

Mortality outcomes	Person‐years	Number of events	Events per 1000 person‐years	Univariable HR	Multivariable adjusted^a^ HR (95% CI)
All‐cause mortality					
Prediagnosis physical activity status					
Inactive	12 4892.4	7067	56.6	Reference	Reference
Active	40 451.6	2027	50.1	0.88 (0.84–0.93)	0.92 (0.88–0.97)
Prediagnosis MET‐min/week (quartiles)					
1. (0 MET‐min/week)	41 642.4	2593	62.3	Reference	Reference
2. (480 ≤ MET‐min/week)	39 580.2	2094	52.9	0.85 (0.80–0.90)	0.95 (0.90–1.01)
3. (930 ≤ MET‐min/week)	42 180.0	2263	53.7	0.86 (0.81–0.91)	0.92 (0.87–0.97)
4. (> 930 MET‐min/week)	41 941.5	2144	51.1	0.82 (0.77–0.87)	0.89 (0.84–0.95)
Lung cancer‐specific mortality					
Prediagnosis physical activity status					
Inactive	124 892.4	5151	41.2	Reference	Reference
Active	40 451.6	1482	36.6	0.89 (0.84–0.94)	0.93 (0.88–0.99)
Prediagnosis MET‐min/week (quartiles)					
1. (0 MET‐min/week)	41 642.4	1901	45.7	Reference	Reference
2. (480 ≤ MET‐min/week)	39 580.2	1494	37.7	0.83 (0.77–0.88)	0.93 (0.87–1.00)
3. (930 ≤ MET‐min/week)	42 180.0	1670	39.6	0.86 (0.81–0.92)	0.92 (0.86–0.99)
4. (> 930 MET‐min/week)	41 941.5	1568	37.4	0.82 (0.76–0.87)	0.90 (0.84–0.96)
Non‐lung cancer mortality					
Prediagnosis physical activity status					
Inactive	124 892.4	1916	15.3	Reference	Reference
Active	40 451.6	545	13.5	0.87 (0.80–0.96)	0.89 (0.81–0.98)
Prediagnosis MET‐min/week (quartiles)					
1. (0 MET‐min/week)	41 642.4	692	16.6	Reference	Reference
2. (480 ≤ MET‐min/week)	39 580.2	600	15.2	0.91 (0.81–1.01)	1.03 (0.92–1.14)
3. (930 ≤ MET‐min/week)	42 180.0	593	14.1	0.85 (0.76–0.94)	0.91 (0.81–1.01)
4. (> 930 MET‐min/week)	41 941.5	576	13.7	0.82 (0.74–0.92)	0.89 (0.79–0.99)

Abbreviations: HR, hazard ratio; MET, metabolic equivalent of task.

^a^ Adjusted for age, sex, body mass index (BMI), smoking status, smoking pack‐year (PY), income, alcohol consumption, and Charlson Comorbidity Index, and lung cancer treatment (receipt of surgery, radiotherapy and/or systemic therapy).

Table 3 presents the association between post‐diagnosis PA and changes in PA status before and after diagnosis with the risk of overall, lung cancer‐specific and non–lung cancer mortality. Compared with inactive individuals at postdiagnosis, physically active individuals showed a significantly reduced risk of all‐cause (aHR = 0.85, 95% CI = 0.81–0.89), lung cancer‐specific (aHR = 0.89, 95% CI = 0.84–0.94) and non–lung cancer (aHR = 0.75, 95% CI = 0.68–0.83) mortality. When stratified by the total amount of post‐diagnosis PA in quartiles, a significant inverse relationship between the amount of PA and mortality risk was observed (Table 3 and Figures 1C,D, S2 and S3). The magnitude of risk reduction for all‐cause, lung cancer‐specific and non–lung cancer mortality with increased PA was higher when assessed with post‐diagnosis PA than that with pre‐diagnosis PA.

**TABLE 3:** Association of postdiagnosis physical activity and changes in physical activity before and after diagnosis with mortality in survivors of lung cancer.

Mortality outcomes	Person‐years	Number of events	Events per 1000 person‐years	Univariable HR	Multivariable adjusted^a^ HR (95% CI)
All‐cause mortality					
Postdiagnosis physical activity status					
Inactive	114 940.9	6838	59.5	Reference	Reference
Active	50 403.1	2256	44.8	0.75 (0.72–0.79)	0.85 (0.81–0.89)
Postdiagnosis MET‐min/week (quartile)					
1. (180 ≤ MET‐min/week)	34 829.0	2589	74.3	Reference	Reference
2. (630 ≤ MET‐min/week)	44 642.6	2353	52.7	0.70 (0.67–0.75)	0.83 (0.78–0.88)
3. (1080 ≤ MET‐min/week)	42 209.8	2179	51.6	0.69 (0.65–0.73)	0.82 (0.77–0.87)
4. (> 1080 MET‐min/week)	43 662.6	1973	45.2	0.60 (0.57–0.64)	0.75 (0.70–0.79)
Change in status between pre‐ and postdiagnosis					
Inactive to inactive	94 376.9	5612	59.5	Reference	Reference
Active to inactive	20 564.0	1226	59.6	1.00 (0.94–1.07)	1.01 (0.95–1.08)
Inactive to active	30 515.5	1455	47.7	0.80 (0.75–0.85)	0.91 (0.85–0.96)
Active to active	19 887.6	801	40.3	0.68 (0.63–0.73)	0.77 (0.71–0.83)
Lung cancer‐specific mortality					
Postdiagnosis physical activity status					
Inactive	114 940.9	4933	42.9	Reference	Reference
Active	50 403.1	1700	33.7	0.79 (0.75–0.83)	0.89 (0.84–0.94)
Postdiagnosis MET‐min/week (quartile)					
1. (180 ≤ MET‐min/week)	34 829.0	1856	53.3	Reference	Reference
2. (630 ≤ MET‐min/week)	44 642.6	1709	38.3	0.72 (0.67–0.77)	0.84 (0.79–0.90)
3. (1080 ≤ MET‐min/week)	42 209.8	1588	37.6	0.70 (0.66–0.75)	0.84 (0.78–0.90)
4. (> 1080 MET‐min/week)	43 662.6	1480	33.9	0.64 (0.59–0.68)	0.79 (0.73–0.84)
Change in status between pre‐ and postdiagnosis					
Inactive to inactive	94 376.9	4054	43.0	Reference	Reference
Active to inactive	20 564.0	879	42.7	0.99 (0.92–1.07)	1.01 (0.94–1.09)
Inactive to active	30 515.5	1097	35.9	0.84 (0.78–0.90)	0.94 (0.88–1.00)
Active to active	19 887.6	603	30.3	0.71 (0.65–0.77)	0.81 (0.75–0.89)
Non‐lung cancer mortality					
Postdiagnosis physical activity status					
Inactive	114 940.9	1905	16.6	Reference	Reference
Active	50 403.1	556	11.0	0.66 (0.60–0.72)	0.75 (0.68–0.83)
Postdiagnosis MET‐min/week (quartile)					
1. (180 ≤ MET‐min/week)	34 829.0	733	21.4	Reference	Reference
2. (630 ≤ MET‐min/week)	44 642.6	644	14.4	0.67 (0.61–0.87)	0.79 (0.71–0.88)
3. (1080 ≤ MET‐min/week)	42 209.8	591	14.0	0.65 (0.59–0.73)	0.77 (0.69–0.86)
4. (> 1080 MET‐min/week)	43 662.6	493	11.3	0.52 (0.47–0.59)	0.65 (0.58–0.74)
Change in status between pre‐ and postdiagnosis					
Inactive to inactive	94 376.9	1588	16.5	Reference	Reference
Active to inactive	20 564.0	347	16.9	1.03 (0.91–1.15)	1.03 (0.92–1.16)
Inactive to active	30 515.5	358	11.7	0.70 (0.62–0.79)	0.83 (0.74–0.93)
Active to active	19 887.6	198	10.0	0.59 (0.51–0.69)	0.66 (0.56–0.76)

Abbreviations: HR, hazard ratio; MET, metabolic equivalent of task.

^a^ Adjusted for age, sex, body mass index (BMI), smoking status, smoking pack‐year (PY), income, alcohol consumption, and Charlson Comorbidity Index, and lung cancer treatment (receipt of surgery, radiotherapy and/or systemic therapy).

### 3.3 Changes in PA Before and After Lung Cancer Diagnosis and Association With Mortality Risk

**FIGURE 2:**
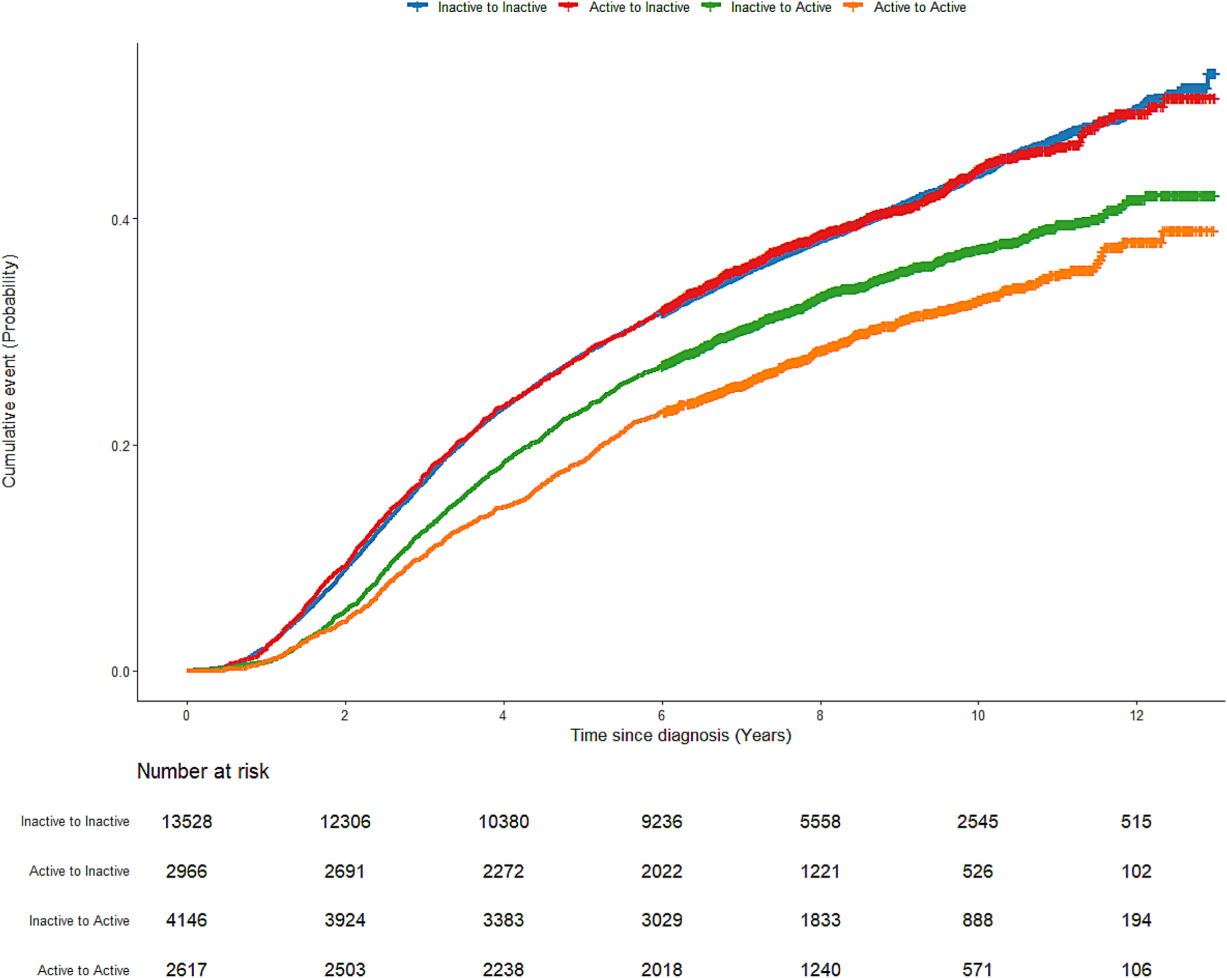
Cumulative incidence of all‐cause mortality in survivors of lung cancer by change in PA before and after diagnosis.

Compared with individuals who were continuously inactive at pre‐ and postdiagnosis of lung cancer, those who were physically active before diagnosis and maintained PA after diagnosis showed the greatest reduction in all‐cause (aHR = 0.77, 95% CI = 0.71–0.83), lung cancer‐specific (aHR = 0.81, 95% CI = 0.75–0.89) and non–lung cancer (aHR = 0.66, 95% CI = 0.56–0.76) mortality. Individuals who were inactive at prediagnosis but promoted PA after lung cancer diagnosis showed a reduced risk of all‐cause (aHR = 0.91, 95% CI = 0.85–0.96), lung cancer‐specific (aHR = 0.94, 95% CI = 0.88–1.00) and non–lung cancer (aHR = 0.83, 95% CI = 0.74–0.93) mortality compared to those who remained inactive after diagnosis. The MCID of PA increase, showing more than 5% all‐cause mortality benefit, was 800 MET‐min/week (aHR = 0.95, 95% CI = 0.92–0.98). However, those who were physically active before diagnosis but became inactive after the diagnosis of lung cancer did not show significantly different survival outcomes compared to those who were continuously inactive (Table 3 and Figures 2, S4 and S5). All results were consistent in the sensitivity analyses, excluding deaths that occurred during the first 2‐year follow‐up (Tables S2 and S3).

### 3.4 Subgroup Analysis of Long‐Term (≥ 5 Years) Survivors of Lung Cancer

The results of the stratified analysis for long‐term survivors (≥ 5 years) of lung cancer are presented in Tables S4 and S5. The significant association of increased pre‐diagnosis and postdiagnosis PA levels with decreased mortality risk remained consistent for all‐cause mortality, with more prominent associations observed when evaluated with post‐diagnosis PA (Figure S6). Compared to individuals who were continuously inactive, a consistently significant reduction in the risk of all‐cause mortality was observed in those who continuously maintained PA before and after diagnosis and in those who promoted PA after lung cancer diagnosis (Figure S7).

## 4 Discussion

In this nationwide population‐based cohort study comprising 23 257 individuals with newly diagnosed lung cancer, we found that both pre‐ and postdiagnosis PA were significantly associated with a reduced risk of death from all causes, lung cancer and non–lung cancer causes. Moreover, the level of pre‐ and postdiagnosis PA showed dose–response associations with reduced mortality risk. Notably, individuals who maintained PA before and after lung cancer diagnosis had the greatest reduction rate (23%) in all‐cause mortality risk compared with those who were consistently inactive. For individuals who were physically inactive at prediagnosis, promoting PA after diagnosis was associated with a 9% reduction in all‐cause mortality risk compared to that in individuals who remained inactive. To the best of our knowledge, this is the first study to investigate the independent associations of pre‐ and postdiagnosis PA with mortality outcomes, specifically evaluating the impact of maintenance and promotion of PA after diagnosis in a large population‐based cohort of survivors of lung cancer.

Given its established benefits in improving health and life expectancy, PA has been an important research target for its potential prognostic effects on mortality in survivors of various cancer types. To date, epidemiologic evidence strongly supports the beneficial effects of PA on survival in patients diagnosed with breast, colon and prostate cancers [11, 34, 35]. Evidence of survival benefits related to PA in these cancer types is corroborated by large observational pan‐cancer studies and meta‐analyses [10, 36]. It has also been integrated into recent exercise guidance for survivors of cancer issued by international organizations, including the ACSM [15, 20], ACS [13] and Clinical Oncology Society of Australia [37]. However, data on survival benefits in lung cancer attributed to PA remain limited. Most studies on this topic have been characterized by small sample sizes or utilizing data derived as part of a pan‐cancer analysis, not specifically focused on lung cancer [36, 38]. Moreover, small sample sizes together with a short duration of follow‐up have resulted in a low number of non‐cancer mortality events. Therefore, all‐cause mortality has rarely been reported in the literature. A recent pan‐cancer meta‐analysis conducted by Friedenreich et al. suggested that postdiagnosis PA levels have a greater impact in reducing both cancer‐specific and all‐cause mortality risks across various cancer sites [36]. When specified for lung cancer, prediagnosis PA decreased the risk of lung cancer‐specific mortality by 19%, and postdiagnosis PA was associated with a 27% decrease in all‐cause mortality risk when comparing the highest and lowest categories of PA levels. However, the effect of prediagnosis PA on all‐cause mortality and postdiagnosis PA on lung cancer‐specific mortality could not be assessed owing to lack of data, even in the setting of a systematic review [36]. Recently, a lung‐cancer‐specific meta‐analysis performed by Yang et al. only reported associations of prediagnosis PA. According to this study, prediagnosis PA was linked to a 7% decrease in the risk of all‐cause mortality, but not to lung cancer‐specific mortality [19]. In a pan‐cancer analysis of patients who participated in the Prostate, Lung, Colorectal, and Ovarian (PLCO) screening trial, postdiagnosis PA revealed no significant association with either cancer‐specific or all‐cause mortality among individuals diagnosed with lung cancer [10]. In addition to limited and often conflicting findings, the lack of studies capturing both pre‐ and postdiagnosis PA within the same population has hindered the ability to assess longitudinal changes in PA and their association with survival outcomes in lung cancer. Consequently, the most recent guidelines from the ACSM Roundtable and ACS were unable to conclude a definitive association between PA and mortality in lung cancer survivors, regarding the absence of comprehensive data on both pre‐ and postdiagnosis PA, as well as all‐cause and cancer‐specific mortality [13, 20]. Our study addresses these gaps by simultaneously evaluating all four key associations, thereby providing novel insights into the importance of changes in PA surrounding the time of diagnosis.

Our study revealed that both pre‐ and postdiagnosis PA had significant protective effects against all‐cause (8% risk reduction related to prediagnosis PA and 15% reduction related to postdiagnosis PA) and cancer‐specific (7% risk reduction related to prediagnosis PA and 11% risk reduction related to postdiagnosis PA) mortality. Moreover, the protective associations were consistently significant for long‐term survivors beyond 5 years. Our findings add novel knowledge to the current literature on the survival benefits attributed to prediagnosis and postdiagnosis PA in survivors of lung cancer, extending previous findings that suggest that postdiagnosis PA levels have a greater impact in reducing mortality risks across various cancer types [36]. In addition, this study found a significant dose–response association of the amount of pre‐ and postdiagnosis PA with reduced mortality risk in survivors of lung cancer. The benefits of increased exercise intensity on mortality have been established in the general population and in certain diseases, including cancer [10, 27, 29, 39], but have been poorly understood in lung cancer.

Moreover, our findings provide the first large‐scale data on how changes in PA levels before and after diagnosis are associated with mortality among survivors of lung cancer. Compared with individuals who were physically inactive before and after diagnosis, those who consistently maintained PA in both conditions showed a 23% reduction in all‐cause mortality, a 19% reduction in lung cancer‐specific mortality and a 34% reduction in non–lung cancer mortality. Notably, individuals who were inactive before lung cancer diagnosis but promoted PA postdiagnosis also showed a significantly reduced risk of all‐cause mortality (by 9%) and lung cancer‐specific mortality (by 6%). Based on our findings, an increase of 800 MET‐min/week can be suggested as a MCID and a potential target for PA promotion after diagnosis to achieve a significant mortality benefit exceeding 5%. However, those who were physically active prediagnosis but failed to maintain PA after diagnosis showed no survival benefits compared with individuals who were consistently inactive. The benefit on survival from maintaining and promoting PA after diagnosis was consistently observed in long‐term (≥ 5 years) survivors. The potential protective biological mechanisms of PA against cancer include modulation of oncogenic factors such as insulin‐like growth factors and inflammatory cytokines, as well as improvement in immunogenicity and metabolic function [14, 40]. These mechanisms can be essential in inhibiting cancer progression and enhancing treatment efficacy [41]. Furthermore, PA has lung‐specific benefits such as protecting pulmonary function and reducing complications related to lung cancer treatment and underlying pulmonary conditions [42, 43]. Our study shows a substantial impact of managing postdiagnosis PA levels on long‐term survival, supporting these mechanisms and providing strong evidence to recommend that individuals with lung cancer increase their PA levels throughout the multiple phases of diagnosis, treatment and post‐treatment.

There are limitations to our study. First, the measures of PA were self‐reported, which may have resulted in recall bias and misclassification of PA levels. Additionally, the use of self‐reported data may be subject to social desirability bias, potentially leading participants to overreport their lifestyle behaviours. Moreover, although our study aimed to evaluate leisure‐time PA as the main exposure, other domains of PA (e.g., occupational or household) could not be accurately captured within our cohort. Second, we included only individuals who survived and were well enough to participate in follow‐up health check‐ups within 2 years after lung cancer diagnosis. While this approach was intended to provide novel data addressing comprehensive associations between PA and lung cancer mortality, it may have led to selection bias disproportionately comprising individuals who were healthier or more health‐conscious. This may limit the generalizability of our findings, particularly among patients with limited life expectancy at diagnosis. Accordingly, the observed long‐term survival benefit associated with maintaining or promoting PA may primarily apply to those healthy enough to endure treatment and participate in health examinations. Third, the NHIS database does not collect data on cancer stages and pathological subtypes of lung cancer. While all analyses were adjusted for available important covariates, the inherent limitation of inability to account for unmeasured prognostic factors may introduce bias, including reverse causation—whereby patients with more advanced disease and poorer performance status are less able to maintain PA and are also at higher risk of mortality. To address this limitation, we conducted subgroup analyses among those who survived at least 2 and 5 years following lung cancer diagnosis. Given that the median 2‐year survival rate for stage IV lung cancer is 10%–23%, and the 5‐year survival rate is as low as 0%–10% [44], the consistent survival benefits observed in these subgroups support the validity of PA as a meaningful factor associated with long‐term survival in individuals with lung cancer. In addition, owing to its observational nature, our findings suggest an association rather than a direct PA‐induced effect. Future randomized controlled trials are warranted to elucidate definite causality.

In conclusion, this nationwide population‐based analysis of individuals newly diagnosed with lung cancer demonstrated that both pre‐ and post‐diagnosis PA were associated with a reduced risk of all‐cause and lung cancer‐specific mortality. A dose–response association was observed between the total amount of PA and its protective effects against death. While those who maintained PA before and after the diagnosis of lung cancer showed the greatest benefit, promoting PA after diagnosis for those who were inactive before also showed significant benefits in terms of mortality reduction. These findings provide clear evidence that encourages individuals with lung cancer to engage in PA for long‐term survival benefits.

## Ethics Statement

This study was approved by the Institutional Review Boards of the Seoul National University Bundang Hospital (No. X‐2203‐747‐901) and the Korean National Health Insurance Service (No. NHIS‐2023‐1‐054).

## Conflicts of Interest

Y.W. Kim has received honorarium from AstraZeneca and research grants not related to this study from AstraZeneca. All other authors declare no conflict of interest.

## Supporting information


**Figure S1:** Flowchart of the study population
**Table S1:** Comparison between included and excluded individuals
**Figure S2:** Cumulative incidence of lung cancer‐specific mortality in survivors of lung cancer by prediagnosis (A: status, B: amount [MET‐min/week] in quartiles) and postdiagnosis physical activity (C: status, D: amount [MET‐min/week] in quartiles)
**Figure S3:** Cumulative incidence of non‐lung cancer mortality in survivors of lung cancer by prediagnosis (A: status, B: amount [MET‐min/week] in quartiles) and postdiagnosis physical activity (C: status, D: amount [MET‐min/week] in quartiles)
**Figure S4:** Cumulative incidence of lung cancer‐specific mortality in survivors of lung cancer by change in PA before and after diagnosis
**Figure S5:** Cumulative incidence of non‐lung cancer mortality in survivors of lung cancer by change in PA before and after diagnosis
**Table S2:** Association between prediagnosis physical activity and mortality in survivors of lung cancer excluding deaths that occurred during the first 2‐year follow‐up
**Table S3:** Association of postdiagnosis physical activity and changes in physical activity before and after diagnosis with mortality in survivors of lung cancer excluding deaths that occurred during the first 2‐year follow‐up
**Table S4:** Association between prediagnosis physical activity and mortality in long‐term (≥ 5 years) survivors of lung cancer
**Table S5:** Association of postdiagnosis physical activity and changes in physical activity before and after diagnosis with mortality in long‐term (≥ 5 years) survivors of lung cancer
**Figure S6:** Cumulative incidence of all‐cause mortality long‐term (≥ 5 years) survivors of lung cancer by prediagnosis (A: status, B: amount [MET‐min/week] in quartiles) and postdiagnosis physical activity (C: status, D: amount [MET‐min/week] in quartiles)
**Figure S7:** Cumulative incidence of all‐cause mortality long‐term (≥ 5 years) survivors of lung cancer by change in PA before and after diagnosis

## Data Availability

The data analysed in this study are not available for public use or sharing because of legislation from the Korean government. However, researchers can apply for the National Health Insurance data‐sharing service upon their IRB approval. Raw data will be available for researchers after a review by the Korea National Health Insurance Sharing Service Institutional Data Access/Ethics Committee.
