# Supplementary material for: Association of Pre‐ and Postdiagnosis Physical Activity, Promotion and Maintenance With Lung Cancer Survival: A Nationwide Cohort Study
Source: J Cachexia Sarcopenia Muscle. 2025 Oct 17;16(5):e70092. doi: 10.1002/jcsm.70092 (PMC12531591; doi:10.1002/jcsm.70092)
Supplement: Supplementary file 1 — Figure S1: Flowchart of the study population Table S1: Comparison between included and excluded individuals Figure S2: Cumulative incidence of lung cancer‐specific mortality in survivors of lung cancer by prediagnosis (A: status, B: amount [MET‐min/week] in quartiles) and postdiagnosis physical activity (C: status, D: amount [MET‐min/week] in quartiles) Figure S3: Cumulative incidence of non‐lung cancer mortality in survivors of lung cancer by prediagnosis (A: status, B: amount [MET‐min/week] in quartiles) and postdiagnosis physical activity (C: status, D: amount [MET‐min/week] in quartiles) Figure S4: Cumulative incidence of lung cancer‐specific mortality in survivors of lung cancer by change in PA before and after diagnosis Figure S5: Cumulative incidence of non‐lung cancer mortality in survivors of lung cancer by change in PA before and after diagnosis Table S2: Association between prediagnosis physical activity and mortality in survivors of lung cancer excluding deaths that occurred during the first 2‐year follow‐up Table S3: Association of postdiagnosis physical activity and changes in physical activity before and after diagnosis with mortality in survivors of lung cancer excluding deaths that occurred during the first 2‐year follow‐up Table S4: Association between prediagnosis physical activity and mortality in long‐term (≥ 5 years) survivors of lung cancer Table S5: Association of postdiagnosis physical activity and changes in physical activity before and after diagnosis with mortality in long‐term (≥ 5 years) survivors of lung cancer Figure S6: Cumulative incidence of all‐cause mortality long‐term (≥ 5 years) survivors of lung cancer by prediagnosis (A: status, B: amount [MET‐min/week] in quartiles) and postdiagnosis physical activity (C: status, D: amount [MET‐min/week] in quartiles) Figure S7: Cumulative incidence of all‐cause mortality long‐term (≥ 5 years) survivors of lung cancer by change in PA before and after diagnosis [file JCSM-16-e70092-s001.docx]

**Association of Pre- and Postdiagnosis Physical Activity, Promotion, and Maintenance with Lung Cancer Survival: A Nationwide Cohort Study**

Yeon Wook Kim, Kyeong Im Kwak, A-Reum Choi, Eung Joo Park, Brian J. Lee, Yeon Joo Lee, Choon-Taek Lee

**Contents**

**Figure S1. Flowchart of the study population**

**Table S1. Comparison between included and excluded individuals**

**Figure S2. Cumulative incidence of lung cancer-specific mortality in survivors of lung cancer by prediagnosis (A: status, B: amount [MET-min/week] in quartiles) and postdiagnosis physical activity (C: status, D: amount [MET-min/week] in quartiles)**

**Figure S3. Cumulative incidence of non-lung cancer mortality in survivors of lung cancer by prediagnosis (A: status, B: amount [MET-min/week] in quartiles) and postdiagnosis physical activity (C: status, D: amount [MET-min/week] in quartiles)**

**Figure S4. Cumulative incidence of lung cancer-specific mortality in survivors of lung cancer by change in PA before and after diagnosis**

**Figure S5. Cumulative incidence of non-lung cancer mortality in survivors of lung cancer by change in PA before and after diagnosis**

**Table S2. Association between prediagnosis physical activity and mortality in survivors of lung cancer excluding deaths that occurred during the first 2-year follow-up**

**Table S3. Association of postdiagnosis physical activity and changes in physical activity before and after diagnosis with mortality in survivors of lung cancer excluding deaths that occurred during the first 2-year follow-up**

**Table S4. Association between prediagnosis physical activity and mortality in long-term (≥5 years) survivors of lung cancer**

**Table S5. Association of postdiagnosis physical activity and changes in physical activity before and after diagnosis with mortality in long-term (≥5 years) survivors of lung cancer**

**Figure S6. Cumulative incidence of all-cause mortality long-term (≥5 years) survivors of lung cancer by prediagnosis (A: status, B: amount [MET-min/week] in quartiles) and postdiagnosis physical activity (C: status, D: amount [MET-min/week] in quartiles)**

**Figure S7. Cumulative incidence of all-cause mortality long-term (≥5 years) survivors of lung cancer by change in PA before and after diagnosis**

**Figure S1. Flowchart of the study population**


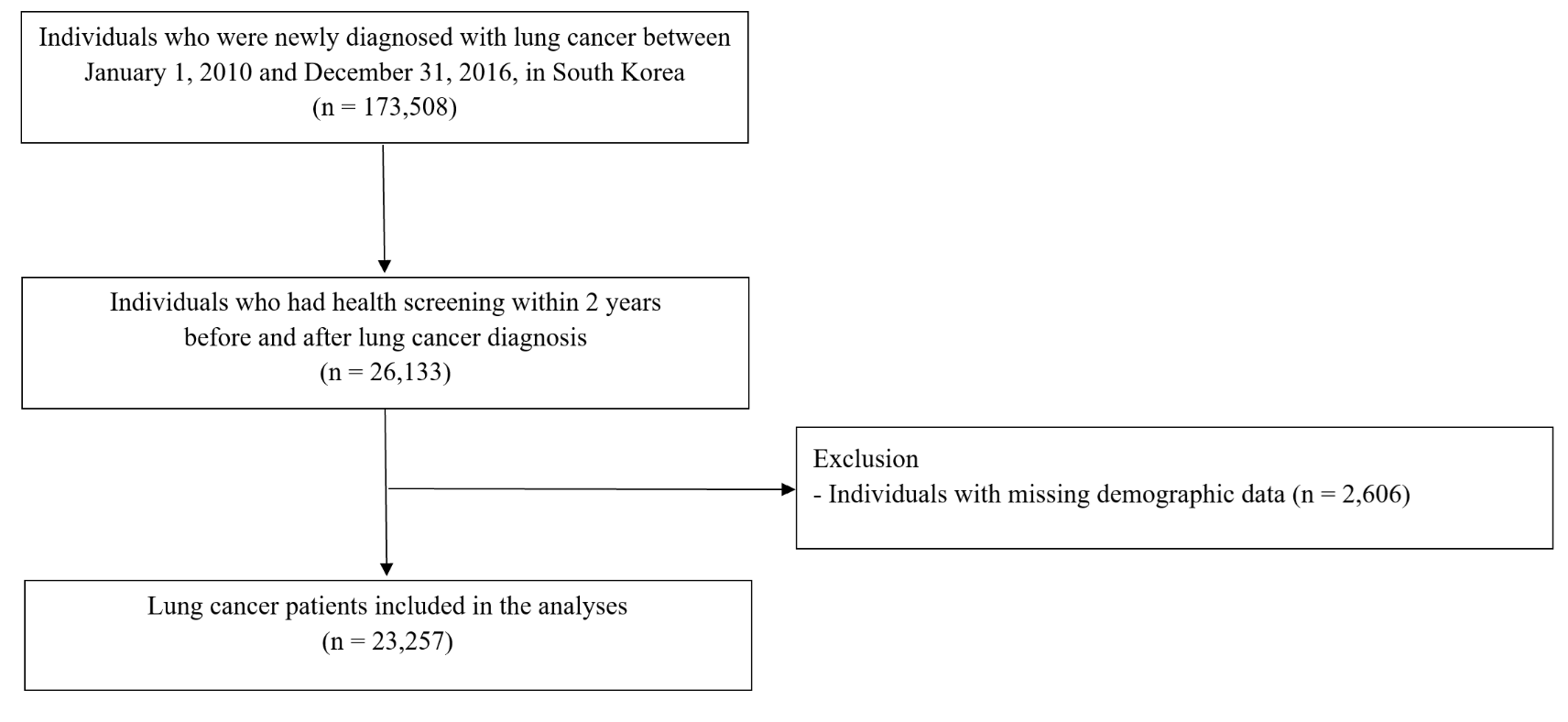


**Table S1. Comparison between included and excluded individuals**

| **Characteristics** | **Included**  **(n=23,257)** | **Excluded**  **(n=150,251)** |
| --- | --- | --- |
| Age ate diagnosis, years, mean ± SD | 63.9 ± 9.9 | 67.2 ± 10.7 |
| Sex, n (%) |  |  |
| Male | 13,936 (59.9) | 77,750 (72.7) |
| Female | 9,321 (40.1) | 29,212 (27.3) |
| Income in quartiles, n (%) |  |  |
| 1~4 or medical aid | 4181 (18.0%) | 15,810 (14.8) |
| 5~8 | 2889 (12.4%) | 13,516 (12.6) |
| 9~12 | 3511 (15.1%) | 17,119 (16.0) |
| 13~16 | 4950 (21.3%) | 23,903 (22.3) |
| 17~20 (highest) | 7726 (33.2%) | 36,614 (34.2) |
| Received treatment, n (%) |  |  |
| Surgery | 15,712 (67.6) | 31,895 (21.2) |
| Radiotherapy | 4,489 (19.3) | 46,010 (30.6) |
| Systematic therapy | 2,172 (9.3) | 24,338 (16.2) |
| 2-year survivors, n (%) | 21,424 (92.1) | 48,398 (32.2) |
| 5-year survivors, n (%) | 17,205 (74.0) | 29,647 (19.7) |

**Figure S2. Cumulative incidence of lung cancer-specific mortality in survivors of lung cancer by prediagnosis (A: status, B: amount [MET-min/week] in quartiles) and postdiagnosis physical activity (C: status, D: amount [MET-min/week] in quartiles)**


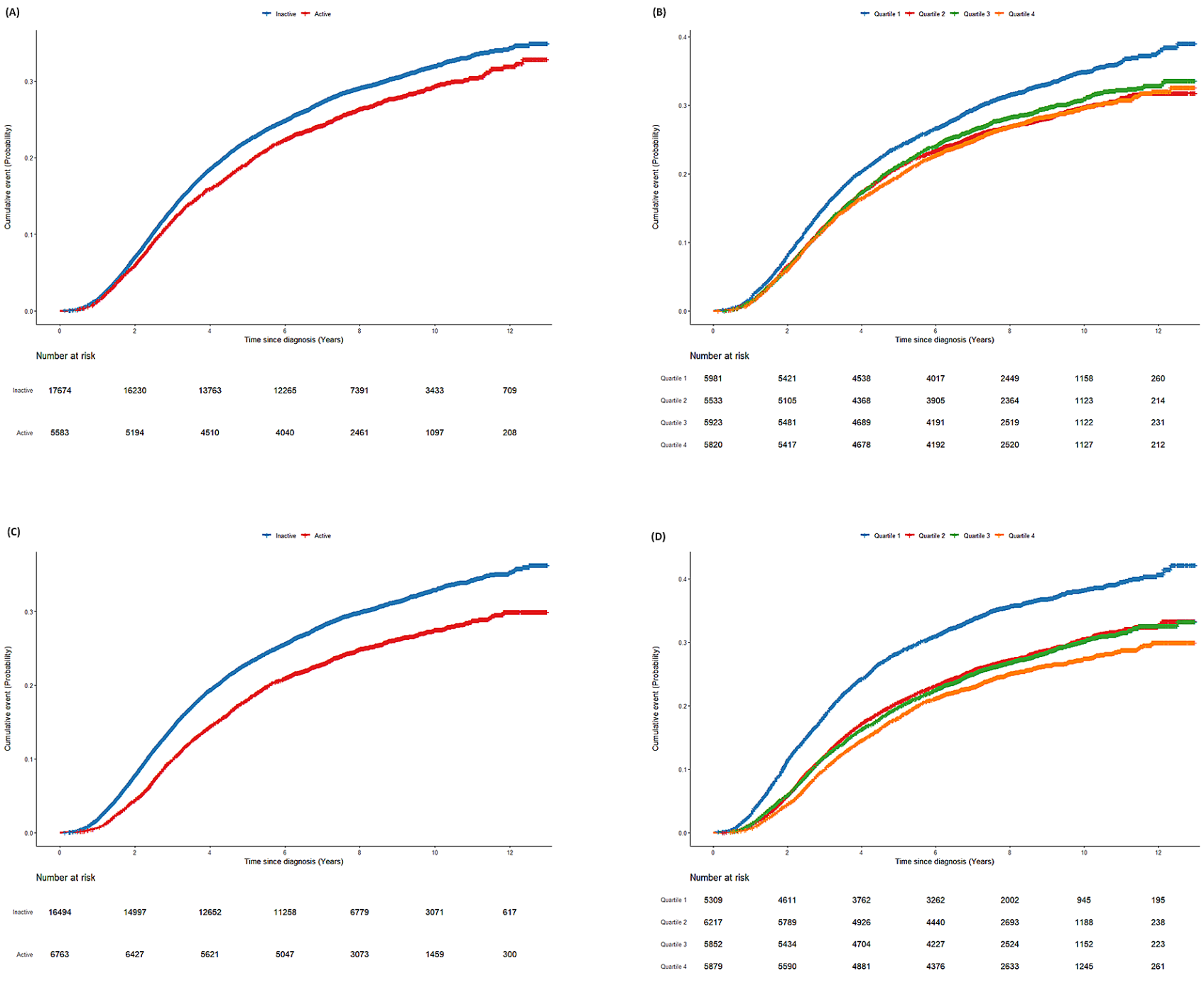


**Figure S3. Cumulative incidence of non-lung cancer mortality in survivors of lung cancer by prediagnosis (A: status, B: amount [MET-min/week] in quartiles) and postdiagnosis physical activity (C: status, D: amount [MET-min/week] in quartiles)**


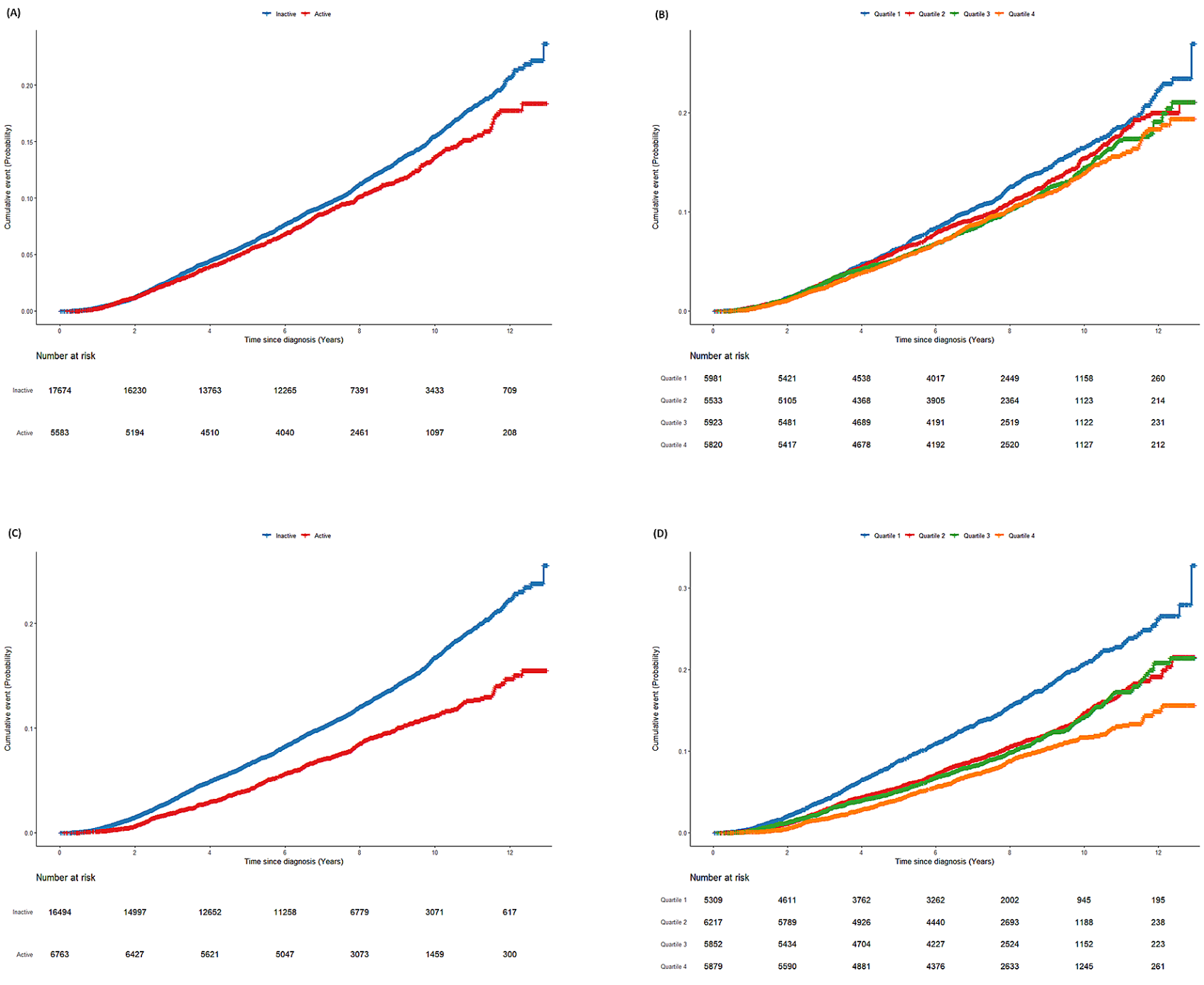


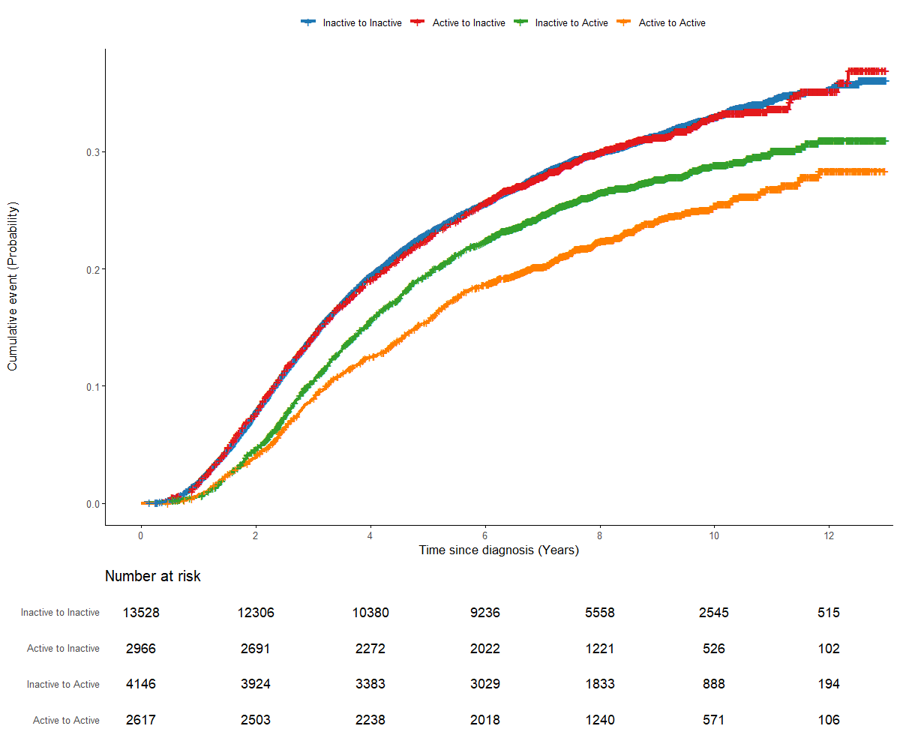
**Figure S4. Cumulative incidence of lung cancer-specific mortality in survivors of lung cancer by change in PA before and after diagnosis**


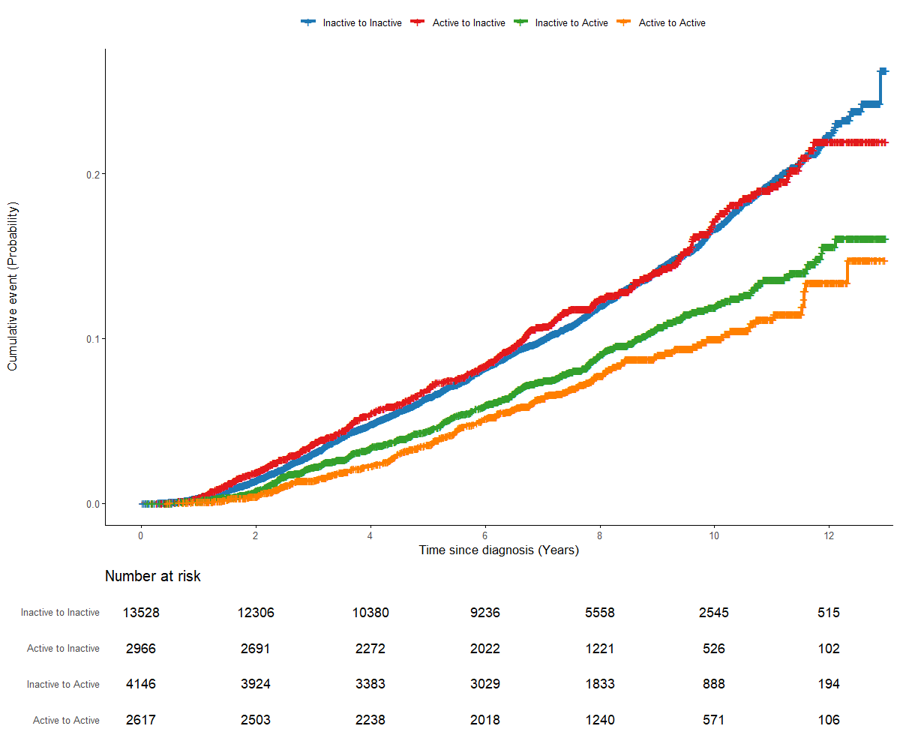
**Figure S5. Cumulative incidence of non-lung cancer mortality in survivors of lung cancer by change in PA before and after diagnosis**

**Table S2. Association between prediagnosis physical activity and mortality in survivors of lung cancer excluding deaths that occurred during the first 2-year follow-up**

| **Mortality outcomes** | **Person-years** | **Number of events** | **Events per**  **1,000 person-years** | **Univariable HR** | **Multivariable adjusted^a^**  **HR (95% CI)** |
| --- | --- | --- | --- | --- | --- |
| All-cause mortality |  |  |  |  |  |
| Prediagnosis physical activity status |  |  |  |  |  |
| Inactive | 122,936.9 | 5,623 | 45.7 | Reference | Reference |
| Active | 39,932.1 | 1,638 | 41.0 | 0.89 (0.85 – 0.94) | 0.92 (0.87 – 0.97) |
| Prediagnosis MET-min/wk (quartiles) |  |  |  |  |  |
| 1 (0 MET-min/wk) | 40,888.3 | 2,033 | 49.7 | Reference | Reference |
| 2 (480≤ MET-min/wk) | 38,999.0 | 1,666 | 42.7 | 0.86 (0.80 – 0.91) | 0.95 (0.89 – 1.02) |
| 3 (930≤ MET-min/wk) | 41,581.1 | 1,821 | 43.8 | 0.88 (0.82 – 0.93) | 0.93 (0.88 – 1.00) |
| 4 ( >930 MET-min/wk) | 41,400.6 | 1,741 | 42.1 | 0.84 (0.79 – 0.90) | 0.91 (0.85 – 0.97) |
| Lung cancer-specific mortality |  |  |  |  |  |
| Prediagnosis physical activity status |  |  |  |  |  |
| Inactive | 122,936.9 | 3,917 | 31.9 | Reference | Reference |
| Active | 39,932.1 | 1,158 | 29.0 | 0.91 (0.85 – 0.97) | 0.95 (0.89 – 1.01) |
| Prediagnosis MET-min/wk (quartiles) |  |  |  |  |  |
| 1 (0 MET-min/wk) | 40,888.3 | 1,418 | 34.7 | Reference | Reference |
| 2 (480≤ MET-min/wk) | 38,999.0 | 1,130 | 29.0 | 0.83 (0.77 – 0.90) | 0.92 (0.85 – 1.00) |
| 3 (930≤ MET-min/wk) | 41,581.1 | 1,300 | 31.3 | 0.90 (0.83 – 0.97) | 0.95 (0.88 – 1.03) |
| 4 ( >930 MET-min/wk) | 41,400.6 | 1,227 | 29.6 | 0.85 (0.79 – 0.92) | 0.92 (0.85 – 1.00) |
| Non-lung cancer mortality |  |  |  |  |  |
| Prediagnosis physical activity status |  |  |  |  |  |
| Inactive | 122,936.9 | 1,706 | 13.9 | Reference | Reference |
| Active | 39,932.1 | 480 | 12.0 | 0.86 (0.78 – 0.95) | 0.87 (0.79 – 0.96) |
| Prediagnosis MET-min/wk (quartiles) |  |  |  |  |  |
| 1 (0 MET-min/wk) | 40,888.3 | 615 | 15.0 | Reference | Reference |
| 2 (480≤ MET-min/wk) | 38,999.0 | 536 | 13.7 | 0.91 (0.81 – 1.02) | 1.03 (0.91 – 1.15) |
| 3 (930≤ MET-min/wk) | 41,581.1 | 521 | 12.5 | 0.84 (0.74 – 0.94) | 0.89 (0.79 – 1.00) |
| 4 ( >930 MET-min/wk) | 41,400.6 | 413 | 12.4 | 0.82 (0.73 – 0.93) | 0.88 (0.78 – 0.99) |

HR, hazard ratio; MET, metabolic equivalent of task

^a^Adjusted for age, sex, body mass index (BMI), smoking status, smoking pack-year (PY), income, alcohol consumption, and Charlson Comorbidity Index, and lung cancer treatment (receipt of surgery, radiotherapy, and/or systemic therapy)

**Table S3. Association of postdiagnosis physical activity and changes in physical activity before and after diagnosis with mortality in survivors of lung cancer excluding deaths that occurred during the first 2-year follow-up**

| **Mortality outcomes** | **Person-years** | **Number of events** | **Events per**  **1,000 person-years** | **Univariable HR** | **Multivariable adjusted^a^**  **HR (95% CI)** |
| --- | --- | --- | --- | --- | --- |
| All-cause mortality |  |  |  |  |  |
| Postdiagnosis physical activity status |  |  |  |  |  |
| Inactive | 112,939.4 | 5,341 | 47.3 | Reference | Reference |
| Active | 49,929.6 | 1,920 | 38.5 | 0.81 (0.77 – 0.85) | 0.90 (0.85 – 0.94) |
| Postdiagnosis MET-min/wk (quartile) |  |  |  |  |  |
| 1 (180≤ MET-min/wk) | 33,913.4 | 1,891 | 55.8 | Reference | Reference |
| 2 (630≤ MET-min/wk) | 44,049.7 | 1,925 | 43.7 | 0.78 (0.73 – 0.83) | 0.89 (0.84 – 0.95) |
| 3 (1,080≤ MET-min/wk) | 41,652.0 | 1,761 | 42.3 | 0.75 (0.70 – 0.80) | 0.87 (0.81 – 0.92) |
| 4 ( >1,080 MET-min/wk) | 43,253.9 | 1,684 | 38.9 | 0.69 (0.65 – 0.74) | 0.83 (0.77 – 0.88) |
| Change in status between  pre- and postdiagnosis |  |  |  |  |  |
| Inactive to Inactive | 92,738.6 | 4,390 | 47.3 | Reference | Reference |
| Active to Inactive | 20,200.8 | 951 | 47.1 | 1.00 (0.93 – 1.07) | 1.00 (0.93 – 1.08) |
| Inactive to Active | 30,198.3 | 1,233 | 40.8 | 0.86 (0.81 – 0.92) | 0.95 (0.90 – 1.02) |
| Active to Active | 19,731.3 | 687 | 34.8 | 0.73 (0.67 – 0.79) | 0.81 (0.75 – 0.88) |
| Lung cancer-specific mortality |  |  |  |  |  |
| Postdiagnosis physical activity status |  |  |  |  |  |
| Inactive | 112,939.4 | 3,669 | 32.5 | Reference | Reference |
| Active | 49,929.6 | 1,406 | 28.2 | 0.86 (0.81 – 0.92) | 0.95 (0.89 – 1.01) |
| Postdiagnosis MET-min/wk  (quartile) |  |  |  |  |  |
| 1 (180≤ MET-min/wk) | 33,913.4 | 1,260 | 37.2 | Reference | Reference |
| 2 (630≤ MET-min/wk) | 44,049.7 | 1,350 | 30.6 | 0.82 (0.76 – 0.89) | 0.93 (0.86 – 1.01) |
| 3 (1,080≤ MET-min/wk) | 41,652.0 | 1,243 | 29.8 | 0.80 (0.74 – 0.86) | 0.92 (0.85 – 0.99) |
| 4 ( >1,080 MET-min/wk) | 43,253.9 | 1,222 | 28.3 | 0.75 (0.70 – 0.82) | 0.89 (0.82 – 0.97) |
| Change in status between  pre- and postdiagnosis |  |  |  |  |  |
| Inactive to Inactive | 92,738.6 | 3,011 | 32.5 | Reference | Reference |
| Active to Inactive | 20,200.8 | 658 | 32.6 | 1.00 (0.92 – 1.09) | 1.02 (0.93 – 1.11) |
| Inactive to Active | 30,198.3 | 906 | 30.0 | 0.92 (0.86 – 0.99) | 1.00 (0.93 – 1.08) |
| Active to Active | 19,731.3 | 500 | 25.3 | 0.78 (0.71 – 0.85) | 0.87 (0.79 – 0.96) |
| Non-lung cancer mortality |  |  |  |  |  |
| Postdiagnosis physical activity status | 112,939.4 | 1,672 | 14.8 | Reference | Reference |
| Inactive | 49,929.6 | 514 | 10.3 | 0.68 (0.62 – 0.76) | 0.78 (0.71 – 0.87) |
| Active |  |  |  |  |  |
| Postdiagnosis MET-min/wk (quartile) | 33,913.4 | 631 | 18.6 | Reference | Reference |
| 1 (180≤ MET-min/wk) | 44,049.7 | 575 | 13.1 | 0.69 (0.62 – 0.78) | 0.81 (0.72 – 0.90) |
| 2 (630≤ MET-min/wk) | 41,652.0 | 518 | 12.4 | 0.66 (0.59 – 0.74) | 0.77 (0.68 – 0.86) |
| 3 (1,080≤ MET-min/wk) | 43,253.9 | 462 | 10.7 | 0.56 (0.50 – 0.63) | 0.70 (0.62 – 0.79) |
| 4 ( >1,080 MET-min/wk) |  |  |  |  |  |
| Change in status between  pre- and postdiagnosis |  |  |  |  |  |
| Inactive to Inactive | 92,738.6 | 1,379 | 14.9 | Reference | Reference |
| Active to Inactive | 20,200.8 | 293 | 14.5 | 0.98 (0.86 – 1.11) | 0.98 (0.87 – 1.11) |
| Inactive to Active | 30,198.3 | 327 | 10.8 | 0.72 (0.64 – 0.81) | 0.85 (0.75 – 0.96) |
| Active to Active | 19,731.3 | 187 | 9.5 | 0.63 (0.54 – 0.73) | 0.68 (0.58 – 0.80) |

HR, hazard ratio; MET, metabolic equivalent of task

^a^Adjusted for age, sex, body mass index (BMI), smoking status, smoking pack-year (PY), income, alcohol consumption, and Charlson Comorbidity Index, and lung cancer treatment (receipt of surgery, radiotherapy, and/or systemic therapy)

**Table S4. Association between prediagnosis physical activity and mortality in long-term (≥5 years) survivors of lung cancer**

| **Mortality outcomes** | **Person-years** | **Number of events** | **Events per**  **1,000 person-years** | **Univariable HR** | **Multivariable adjusted^a^**  **HR (95% CI)** |
| --- | --- | --- | --- | --- | --- |
| All-cause mortality |  |  |  |  |  |
| Prediagnosis physical activity status |  |  |  |  |  |
| Inactive | 111,960.2 | 2,326 | 20.8 | Reference | Reference |
| Active | 36,877.1 | 716 | 19.4 | 0.94 (0.86 – 1.02) | 0.95 (0.87 – 1.03) |
| Prediagnosis MET-min/wk (quartiles) |  |  |  |  |  |
| 1 (0 MET-min/wk) | 37,056.3 | 871 | 23.5 | Reference | Reference |
| 2 (480≤ MET-min/wk) | 35,604.8 | 661 | 18.6 | 0.79 (0.71 – 0.88) | 0.88 (0.79 – 0.97) |
| 3 (930≤ MET-min/wk) | 38,026.5 | 755 | 19.9 | 0.86 (0.78 – 0.94) | 0.91 (0.82 – 1.00) |
| 4 ( >930 MET-min/wk) | 38,149.8 | 755 | 19.8 | 0.85 (0.77 – 0.94) | 0.91 (0.82 – 1.00) |
| Lung cancer-specific mortality |  |  |  |  |  |
| Prediagnosis physical activity status |  |  |  |  |  |
| Inactive | 111,960.2 | 1,321 | 11.8 | Reference | Reference |
| Active | 36,877.1 | 435 | 11.8 | 1.00 (0.90 – 1.12) | 1.04 (0.93 – 1.15) |
| Prediagnosis MET-min/wk (quartiles) |  |  |  |  |  |
| 1 (0 MET-min/wk) | 37,056.3 | 502 | 13.5 | Reference | Reference |
| 2 (480≤ MET-min/wk) | 35,604.8 | 360 | 10.1 | 0.75 (0.65 – 0.85) | 0.82 (0.71 – 0.93) |
| 3 (930≤ MET-min/wk) | 38,026.5 | 443 | 11.7 | 0.87 (0.76 – 0.98) | 0.92 (0.81 – 1.05) |
| 4 ( >930 MET-min/wk) | 38,149.8 | 451 | 11.8 | 0.88 (0.77 – 1.00) | 0.94 (0.83 – 1.07) |
| Non-lung cancer mortality |  |  |  |  |  |
| Prediagnosis physical activity status |  |  |  |  |  |
| Inactive | 111,960.2 | 1,005 | 9.0 | Reference | Reference |
| Active | 36,877.1 | 281 | 7.6 | 0.85 (0.75 – 0.98) | 0.85 (0.74 – 0.97) |
| Prediagnosis MET-min/wk (quartiles) |  |  |  |  |  |
| 1 (0 MET-min/wk) | 37,056.3 | 369 | 10.0 | Reference | Reference |
| 2 (480≤ MET-min/wk) | 35,604.8 | 301 | 8.5 | 0.85 (0.73 – 0.99) | 0.97 (0.83 – 1.13) |
| 3 (930≤ MET-min/wk) | 38,026.5 | 312 | 8.2 | 0.84 (0.72 – 0.98) | 0.89 (0.77 – 1.04) |
| 4 ( >930 MET-min/wk) | 38,149.8 | 304 | 8.0 | 0.82 (0.70 – 0.95) | 0.86 (0.74 – 1.00) |

HR, hazard ratio; MET, metabolic equivalent of task

^a^Adjusted for age, sex, body mass index (BMI), smoking status, smoking pack-year (PY), income, alcohol consumption, and Charlson Comorbidity Index, and lung cancer treatment (receipt of surgery, radiotherapy, and/or systemic therapy)

**Table S5. Association of postdiagnosis physical activity and changes in physical activity before and after diagnosis with mortality in long-term (≥5 years) survivors of lung cancer**

| **Mortality outcomes** | **Person-years** | **Number of events** | **Events per**  **1,000 person-years** | **Univariable HR** | **Multivariable adjusted^a^**  **HR (95% CI)** |
| --- | --- | --- | --- | --- | --- |
| All-cause mortality |  |  |  |  |  |
| Postdiagnosis physical activity status |  |  |  |  |  |
| Inactive | 102,618.9 | 2,227 | 21.7 | Reference | Reference |
| Active | 46,218.4 | 815 | 17.6 | 0.81 (0.74 – 0.87) | 0.88 (0.81 – 0.96) |
| Postdiagnosis MET-min/wk (quartile) |  |  |  |  |  |
| 1 (180≤ MET-min/wk) | 30,104.1 | 749 | 24.9 | Reference | Reference |
| 2 (630≤ MET-min/wk) | 40,360.1 | 802 | 19.9 | 0.80 (0.73 – 0.89) | 0.90 (0.81 – 0.99) |
| 3 (1,080≤ MET-min/wk) | 38,372.4 | 777 | 20.2 | 0.82 (0.74 – 0.91) | 0.92 (0.83 – 1.02) |
| 4 ( >1,080 MET-min/wk) | 40,000.8 | 714 | 17.9 | 0.72 (0.65 – 0.80) | 0.84 (0.75 – 0.93) |
| Change in status between  pre- and postdiagnosis |  |  |  |  |  |
| Inactive to Inactive | 84,228.5 | 1,827 | 21.7 | Reference | Reference |
| Active to Inactive | 18,390.4 | 400 | 21.8 | 1.01 (0.91 – 1.13) | 1.01 (0.91 – 1.13) |
| Inactive to Active | 27,731.7 | 499 | 18.0 | 0.82 (0.75 – 0.91) | 0.91 (0.83 – 1.00) |
| Active -> Active | 18,486.7 | 316 | 17.1 | 0.79 (0.70 – 0.88) | 0.84 (0.75 – 0.95) |
| Lung cancer-specific mortality |  |  |  |  |  |
| Postdiagnosis physical activity status |  |  |  |  |  |
| Inactive | 102,618.9 | 1.250 | 12.2 | Reference | Reference |
| Active | 46,218.4 | 506 | 10.9 | 0.90 (0.81 – 0.99) | 0.96 (0.86 – 1.06) |
| Postdiagnosis MET-min/wk  (quartile) |  |  |  |  |  |
| 1 (180≤ MET-min/wk) | 30,104.1 | 402 | 13.4 | Reference | Reference |
| 2 (630≤ MET-min/wk) | 40,360.1 | 463 | 11.5 | 0.86 (0.75 – 0.98) | 0.95 (0.83 – 1.09) |
| 3 (1,080≤ MET-min/wk) | 38,372.4 | 454 | 11.8 | 0.89 (0.78 – 1.02) | 0.98 (0.86 – 1.13) |
| 4 ( >1,080 MET-min/wk) | 40,000.8 | 437 | 10.9 | 0.82 (0.72 – 0.94) | 0.93 (0.81 – 1.07) |
| Change in status between  pre- and postdiagnosis |  |  |  |  |  |
| Inactive to Inactive | 84,228.5 | 1,017 | 12.1 | Reference | Reference |
| Active to Inactive | 18,390.4 | 233 | 12.7 | 1.06 (0.92 – 1.22) | 1.07 (0.92 – 1.23) |
| Inactive to Active | 27,731.7 | 304 | 11.0 | 0.91 (0.80 – 1.03) | 0.96 (0.85 – 1.10) |
| Active to Active | 18,486.7 | 202 | 10.9 | 0.90 (0.78 – 1.05) | 0.98 (0.84 – 1.14) |
| Non-lung cancer mortality |  |  |  |  |  |
| Postdiagnosis physical activity status |  |  |  |  |  |
| Inactive | 102,618.9 | 977 | 9.5 | Reference | Reference |
| Active | 46,218.4 | 309 | 6.7 | 0.69 (0.61 – 0.79) | 0.78 (0.69 – 0.89) |
| Postdiagnosis MET-min/wk (quartile) |  |  |  |  |  |
| 1 (180≤ MET-min/wk) | 30,104.1 | 347 | 11.5 | Reference | Reference |
| 2 (630≤ MET-min/wk) | 40,360.1 | 339 | 8.4 | 0.73 (0.63 – 0.85) | 0.84 (0.72 – 0.97) |
| 3 (1,080≤ MET-min/wk) | 38,372.4 | 323 | 8.4 | 0.74 (0.64 – 0.86) | 0.85 (0.73 – 0.99) |
| 4 ( >1,080 MET-min/wk) | 40,000.8 | 277 | 6.9 | 0.60 (0.51 – 0.71) | 0.73 (0.62 – 0.86) |
| Change in status between  pre- and postdiagnosis |  |  |  |  |  |
| Inactive to Inactive | 84,228.5 | 810 | 9.6 | Reference | Reference |
| Active to Inactive | 18,390.4 | 167 | 9.1 | 0.95 (0.81 – 1.13) | 0.95 (0.80 – 1.12) |
| Inactive to Active | 27,731.7 | 195 | 7.0 | 0.72 (0.62 – 0.84) | 0.85 (0.73 – 1.00) |
| Active to Active | 18,486.7 | 114 | 6.2 | 0.64 (0.52 – 0.78) | 0.68 (0.56 – 0.82) |

HR, hazard ratio; MET, metabolic equivalent of task

^a^Adjusted for age, sex, body mass index (BMI), smoking status, smoking pack-year (PY), income, alcohol consumption, and Charlson Comorbidity Index, and lung cancer treatment (receipt of surgery, radiotherapy, and/or systemic therapy)

**Figure S6. Cumulative incidence of all-cause mortality long-term (≥5 years) survivors of lung cancer by prediagnosis (A: status, B: amount [MET-min/week] in quartiles) and postdiagnosis physical activity (C: status, D: amount [MET-min/week] in quartiles)**


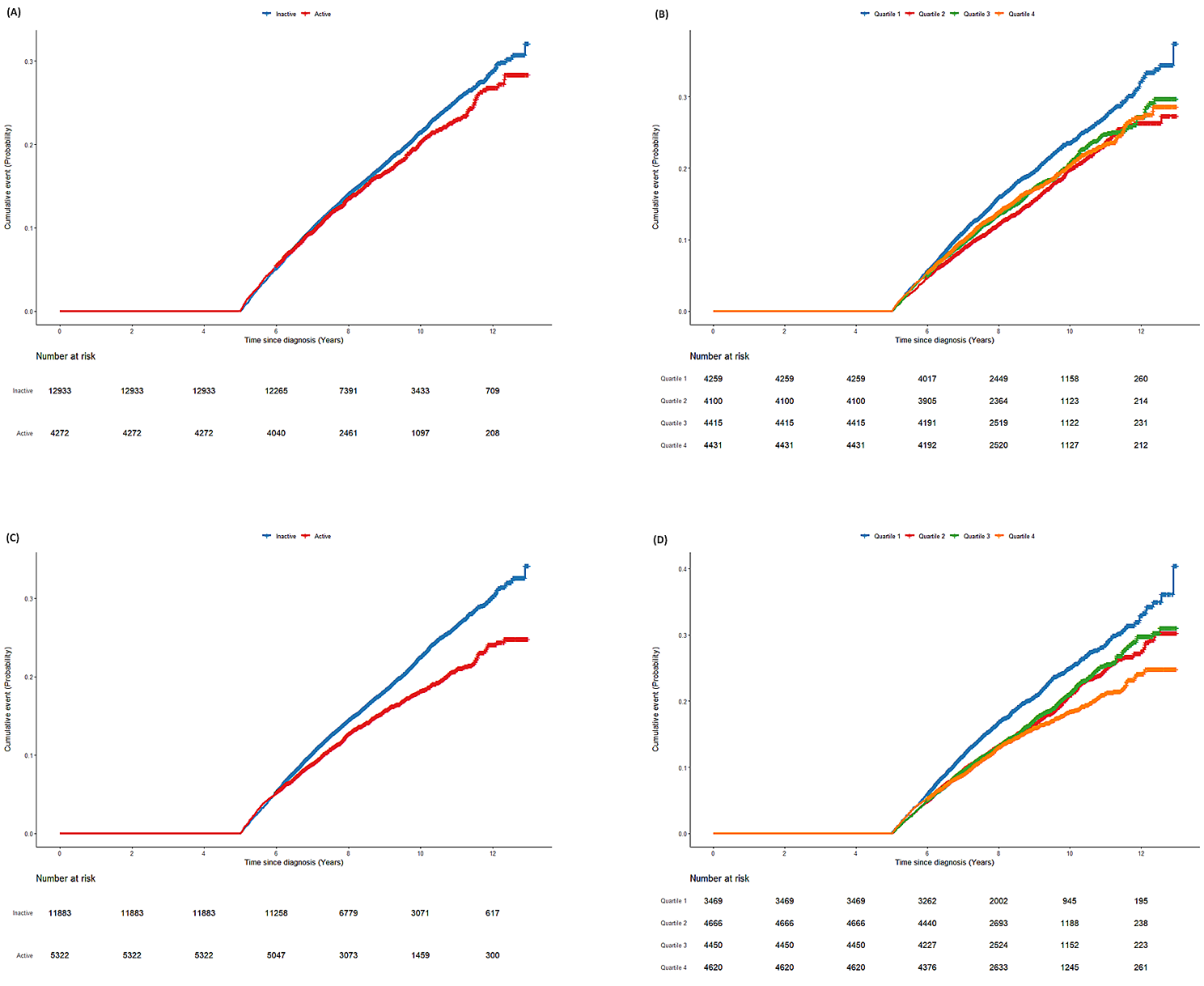


**
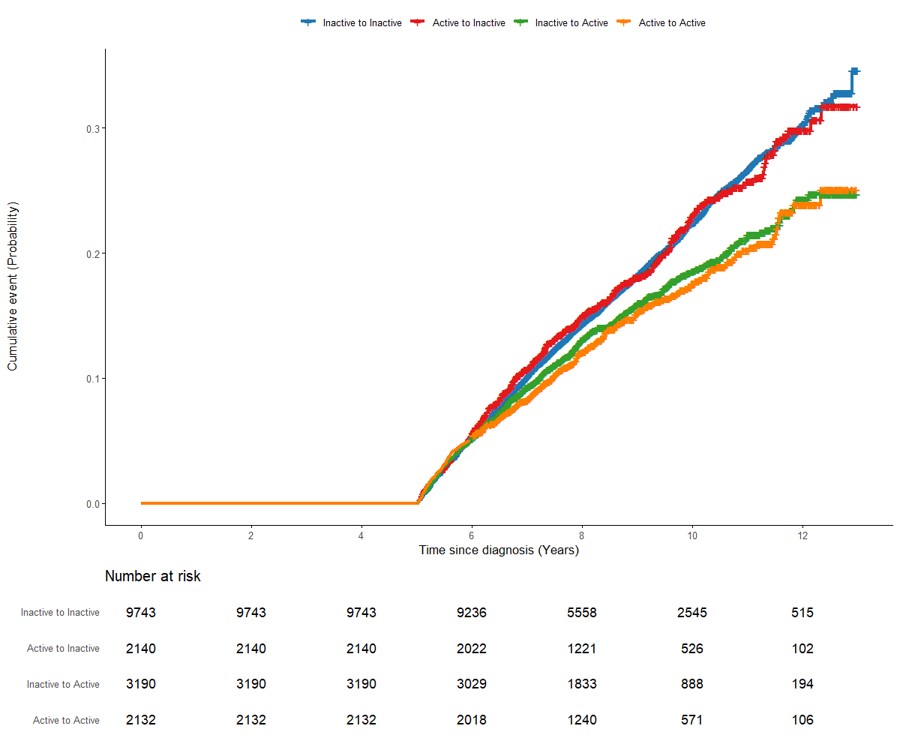
Figure S7. Cumulative incidence of all-cause mortality long-term (≥5 years) survivors of lung cancer by change in PA before and after diagnosis**
